# GTP-Binding Protein 1-Like (GTPBP1l) Regulates Vascular Patterning during Zebrafish Development

**DOI:** 10.3390/biomedicines10123208

**Published:** 2022-12-10

**Authors:** Yi-Hao Lo, Yi-Shan Huang, Yu-Chiuan Chang, Pei-Yu Hung, Wen-Der Wang, Wangta Liu, Ritesh Urade, Zhi-Hong Wen, Chang-Yi Wu

**Affiliations:** 1 Department of Family Medicine, Zouying Branch of Kaohsiung Armed Forces General Hospital, Kaohsiung 813, Taiwan; 2 Department of Marine Biotechnology and Resources, National Sun Yat-sen University, Kaohsiung 804, Taiwan; 3 Institute of Medical Science and Technology, National Sun Yat-sen University, Kaohsiung 804, Taiwan; 4 Department of Biological Sciences, National Sun Yat-sen University, Kaohsiung 804, Taiwan; 5 Doctoral Degree Program in Marine Biotechnology, National Sun Yat-sen University, Kaohsiung 804, Taiwan; 6 Institute of Biomedical Sciences, National Sun Yat-sen University, Kaohsiung 804, Taiwan; 7 Department of Bioagricultural Science, National Chiayi University, Chiayi 600, Taiwan; 8 Department of Biotechnology, Kaohsiung Medical University, Kaohsiung 807, Taiwan

**Keywords:** *gtpbp1l*, vascular development, ISV (intersegmental vessel), CVP (caudal vein plexus), zebrafish

## Abstract

Genetic regulation of vascular patterning is not fully understood. Here, we report a novel gene, *gtpbp1l* (*GTP-binding protein 1-like*), that regulates vascular development in zebrafish. Amino acid sequence comparison and a phylogenetic study showed that *gtpbp1l* is conserved in vertebrates. *Gtpbp1l* mRNA is expressed in the vasculature during embryogenesis. Knockdown of *gtpbp1l* by morpholino impairs the patterning of the intersegmental vessel (ISV) and caudal vein plexus (CVP), indicating the role of *gtpbp1l* in vasculature. Further apoptosis assays and transgenic fish tests suggested that vascular defects in *gtpbp1l* morphants are not due to cell death but are likely caused by the impairment of migration and proliferation. Moreover, the altered expression of vessel markers is consistent with the vascular defects in *gtpbp1l* morphants. Finally, we revealed that *gtpbp1l* is regulated by VEGF/notch and BMP signaling. Collectively, these findings showed that *gtpbp1l* plays a critical role in vascular patterning during zebrafish development.

## 1. Introduction

The right patterning and function of the vascular network in vertebrates are essential for delivering nutrients or oxygen and removing waste. During embryogenesis, failure to form a properly functioning vasculature causes embryonic lethality [1]. The de novo formation of new vessels, termed vasculogenesis and angiogenesis, is a process in which additional vessels sprout and grow from preexisting vascular structures [2].

Zebrafish are gaining popularity among other vertebrate models in various fields, addressing mechanisms relative to blood vessel development including angioblast specification [3], artery-veinous specification [4], and patterning and morphogenesis [5,6,7]. Angioblast progenitors specify arteries and veins primarily via VEGF–Notch signaling interplay. Further, angioblasts sprout, proliferate, and migrate from the preexisting main vessels to form a secondary vessel network, such as intersegmental vessels (ISVs) in the zebrafish trunk. The timing and directions are mediated by several molecules during the growth of ISVs [8,9]. However, these mediators have not been completely characterized with respect to angiogenesis during the development of embryos. One of the endothelial progenitor cells called the tip cell receives and follows guidance cues, while the other stalk cells trail up and form a lumen [10]. Previously, several pathways were linked to tip–stalk cell specification, which includes Notch and VEGFC/VEGR3 [10,11]. The regulators and molecular signals that govern the specification of tip and stalk cells are still vague. In addition to the angiogenesis of ISV, endothelial progenitor cells also sprouted ventrally from the posterior cardinal vein to form a honeycomb-like vessels network called the caudal vein plexus (CVP) via BMP signal pathways [12,13]. However, their regulators and mechanism have largely remained uncertain.

GTPases are key proteins in many critical biological processes. The small GTPase Rap1 has been shown to promote VEGFR2 activation and angiogenesis [14]. Members of the Ras GTPase family have been shown to function in vascular patterning via semaphorin-Plexin signaling [15]. The G-protein-coupled receptor GPCR126 is involved in pathological and developmental angiogenesis by regulating VEGFR2 signals [16]. In addition to typical small GTPases and GPCRs, the GTP-binding protein superfamily includes the putative GTPase encoded by the *Gtpbp1l* (GTP binding protein 1-like) gene. The encoded amino acid sequence of this protein showed the highest homology with human GTPBP1. GTPBP1 possesses eEF1A-like elongation activity, delivering aa-tRNA to the A site of the ribosome in a GTP-dependent manner. Thus, GTPBP1 is classified as a translational GTPase (trGTPase) superfamily [17]. Furthermore, GTPBP1 has been shown to interact with exosomes and can stimulate the exosomal degradation of mRNAs engaged within elongation complexes [18]. A recent study showed that tRNA deficiency leads to ribosomal arresting and neurodegeneration and GTPBP1 resolves stalled ribosomes to regulate neuronal homeostasis during defective elongation [19]. However, there have been no reports on the function of the GTPBP superfamily in vessels.

Here, we report a novel *gtpbp1l* gene that controls vascular development in zebrafish. We showed that *Gtpbp1l* mRNA is expressed in developing vessels, and a loss of *gtpbp1l* impairs the patterning of ISV and CVP, indicating the role of *gtpbp1l* in vasculature. We further showed vascular defects are likely caused by the impairment of migration and proliferation. Furthermore, the reduced expression of vessel genes is consistent with the vascular defects in *gtpbp1l* morphants. We also revealed that *gtpbp1l* is regulated by VEGF/notch and BMP signals. Together, these findings showed that *gtpbp1l* plays a critical role in vascular development.

## 2. Materials and Methods

### 2.1. Zebrafish and Chemical Treatments

Zebrafish were raised at 28.5 °C as recommended [20] and managed according to regulations referred by the Institutional Animal Care Committee NSYSU (IACUC no. 10752). Wild-type AB or TL fish and transgenic lines *Tg(kdrl:eGFP)^la116^*, *Tg(kdrl:mCherry)^ci5^,* and *Tg(fli1a:negfp)^y7^* [21] were obtained from the Zebrafish Core Facility in Taiwan. Furthermore, 0.003% 1-phenyl-2-thiourea (PTU, Sigma, St. Louis, MO, USA) was added to the fish medium to prevent pigment formation of embryos at 6 h post-fertilization (hpf). DAPT, SU5416, Dorsomorphin (DM), and DMH1 (Sigma) were used to inhibit the respective pathways, and DMSO was used as the control.

### 2.2. Morpholino and mRNA Injection

Microinjections were performed with borosilicate filament glass microcapillaries that were pulled with a P-97 micropipette puller (Sutter Instrument, Novato, CA, USA). Morpholinos (MOs) were obtained from the Gene-Tools company (Philomath, OR, USA). Morpholinos were designed to block the translation of *gtpbp1l* by targeting the first codon (*gtpbp1l^atg^ MO*) or disrupt the splicing of *gtpbp1l* (*gtpbp1l ^e4i4^MO*) by targeting the exon 4-intron 4 boundary; the MO sequences were as follows:

*gtpbp1l ^atg^MO*: 5′-GCTGCTTCAGCATCTGTCTGGAAAA-3′;

*gtpbp1l ^e4i4^MO*: 5′-GCACACCTACTGCCTCTCACCATTA-3′.

The quantities of morpholinos injected were 3.4 ng and 8 ng, respectively. For rescue experiments, we generated a *gtpbp1l* mRNA expression construct using Tol2kit vectors with the multisite Gateway cloning system (Invitrogen, Philadelphia, PA, USA). The *gtpbp1l*-coding region flanking the *attb1/b2* sequences was amplified from cDNA by using the primers listed in Table S1 to generate pDONR-*gtpbp1l*. Capped and polyadenylated *gtpbp1l* mRNA was synthesized in vitro using the mMESSAGE mMACHINE transcription kit with SP6 RNA polymerase (Ambion, Austin, TX, USA). All injections were performed on one-cell zebrafish embryos by using a FemtoJet microinjector (Eppendorf, Hamburg, Germany).

### 2.3. RNA Extraction and RT–qPCR Assays

Total RNA was isolated using the RNeasy kit (Qiagen, Valencia, CA, USA) and mRNA was reverse transcribed to cDNA using the oligo-dT primer (Roche Applied Science, Branford, CT, USA) based on the manufacturer’s protocols. Quantitative PCR was performed using reagent SYBR Green Mix (Roche), and qPCR primers used in this study are listed in Table S1. The relative expression of respective genes was analyzed and calculated by the ΔΔCt method by considering *β-actin* as an internal control. All biological experiments were performed in triplicate.

### 2.4. Whole-Mount In-Situ Hybridization and Cryosectioning

The in situ probe generation for *ephrinb2, mrc1, flt4,* and *stabilin* was described previously [22]. The *gtpbp1l* 0.5 kb fragment was obtained by PCR amplification using the primers listed below and the PCR product was cloned into the pGMT Easy vector (Promega, Madison, WI, USA). The plasmids were linearized and subjected to in vitro transcription using T7 or SP6 RNA Polymerase (Roche) with a DIG-labeled RNA labeling kit (Roche) to synthesize antisense and sense probes, respectively, for in situ hybridization.

*gtpbp1l*-f: 5′-GCAGTCCAGCAAGAAACCTCC-3′

*gtpbp1l*-r: 5′-CGGTTTTATCCACCGTGATT-3′

Briefly, regarding the hybridization procedure, the embryos were fixed using 4% paraformaldehyde (PFA) and stored at −20 °C in methanol for dehydration until further use. After cycles of rehydration, embryos were permeabilized with 10 µg/mL of Proteinase K (Roche). Then the embryos were incubated with respective probes at 65°C overnight and blocked with 1% BSA for 2 h. Embryos were then incubated with AP-conjugated anti-DIG antibody overnight at 4 °C. After several wash steps to remove extra antibodies, an NBT and BCIP substrate (Roche) was used for color development. Embryos were photographed via embedding in 3% methylcellulose. For cryo-sectioning, embryos were fixed with the TekOCT tissue freezing medium, sectioned at approximately 10 µm thickness using a Leica CM3050S cryostat (Leica Biosystems, Deer Park, TX, USA), and photographed.

### 2.5. Image Processing and Analysis

Methyl cellulose (Sigma) or low-melting agarose (Invitrogen) are used for embryo embedding. White-light or fluorescent images were photographed with a high-resolution camera Axiocam (Carl Zeiss, Jena, Germany) on the Lumar V12 stereomicroscope and photos were processed by AxioVision software. For the confocal photos, embryos were embedded in low-melting agarose with 5% tricaine to immobilize them, and images were collected on Zeiss LSM700 confocal microscopes and processed in ZEN software (Carl Zeiss) or ImageJ software (NIH, Bethesda, MD, USA).

### 2.6. TUNEL Assay

The protocol of the TUNEL assay for apoptotic cell detection is based on a previous publication [22,23]. Briefly, embryos were fixed in PFA and dehydrated in methanol until use. After rehydration and permeabilization, apoptotic cells were detected by a TUNEL assay kit (Roche) according to the manufacturer’s instructions. The fixed embryos were treated with 3% H_2_O_2_, washed, and labeled with the TUNEL enzyme. After being washed to remove extra non-incorporated nucleotides and blocked with 5% sheep serum, embryos were then incubated with the peroxidase (POD)-conjugated anti-fluorescein antibody (Roche) overnight at 4°C. Color development was visualized using a DAB kit (Roche).

### 2.7. Acridine Orange Staining

Embryos were dechorionated and cultured in a fish medium containing 2 µg/mL acridine orange (AO, Sigma) for 40 min. To remove extra acridine orange, embryos were washed six times with a fresh fish medium. The embryos were then embedded in 3% methylcellulose and photographed.

### 2.8. Statistical Analysis

Data are presented as the mean ± standard deviation (SD). A one-way analysis of variance (ANOVA) was used to compare multiple groups. The difference between the two groups was evaluated using Student’s t-test. *p* values < 0.05 were considered statistically significant.

## 3. Results

### 3.1. Gtpbp1l Is Expressed in Developing Vessels

**Figure 1:**
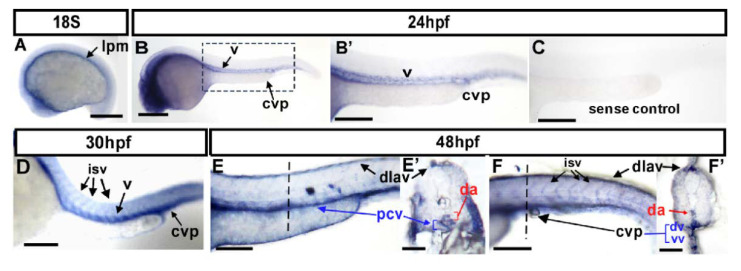
Expression pattern of *gtpbp1l* mRNA during zebrafish vessel development. (**A**–**F**) Spatiotemporal expression of *gtpbp1l* in the vessels (v), caudal vein plexus (CVP), intersegmental vessels (isv), and dorsal longitudinal anastomotic vessel (dlav) during development as shown at 18S, 24 hpf, 30 hpf, and 48 hpf. A cross-section taken at 48 hpf (**E’**,**F’**) clearly shows the expression of *gtpbp1l* in the dorsal aorta (da), posterior vein (pcv), and CVP. (**A**) At the 18 somite (18S) stage, *gtpbp1l* expression is in the lateral plate mesoderm (lpm). (**B**,**B’**) At 24 hpf, *gtpbp1l* is expressed in the vessels (v) and caudal vein plexus (CVP). (**B’**) is an enlargement of (**B**). (**C**) The *gtpbp1l* sense probe served as a negative control. (**D**) At 30 hpf, *gtpbp1l* expression can be observed in the vessels, isv, and CVP (**E**,**E’**,**F**,**F’**) At 48 hpf, *gtpbp1l* is expressed continuously in the vessels, isv, dlav, and CVP at the region of the trunk (**E**) and tail (**F**). (**E’**,**F’**) Cross-sections of embryos from (**E**,**F**) show that *gtpbp1l* is expressed in the dorsal aorta (da), posterior cardinal vein (pcv), dorsal vein (dv), and ventral vein (vv) of the CVP. Scale bars are 200 µm.

We are interested in identifying molecules that are required for vascular patterning. The *gtpbp1l* (*GTP binding protein 1-like*) gene was identified as a potential target in our previous transcriptome screening for genes regulated by the transcription factor islet2/nr2f1b [24]. *Gtpbp1l* encodes a putative GTPase and belongs to the GTP-binding protein superfamily. Its deduced amino acid sequence exhibited the highest overall homology with human GTPBP1 (Figure S1). To determine *gtpbp1l’s* role in zebrafish vasculature, we performed whole-mount in situ hybridization. At the 18 somite stage (S), it is expressed in the eyes, brain, head, and lateral plate mesoderm (lpm) (Figure 1A). Expression at the lpm corresponds to its localization in developing vessels. At 24 hpf, it is expressed in the eyes, brain, vessels (v), and caudal vein plexus (CVP) in the trunk (Figure 1B,B’). At 30 and 48 hpf, it continued to be expressed in the trunk, especially in the vessels, intersegmental vessels (ISVs), dorsal longitudinal anastomotic vessels (DLAV), and CVP (Figure 1D–F). Transverse sections of the 48 hpf embryo further confirm its expression in primary vessels such as the dorsal aorta (DA), posterior cardinal vein (PCV), and CVP (Figure 1E’,F’). These data show the expression of *gtpbp1l* in developing vessels and suggest the function of *gtpbp1l* in vascular development.

### 3.2. Knockdown of gtpbp1l Causes Vascular Defects during Zebrafish Embryogenesis

**Figure 2:**
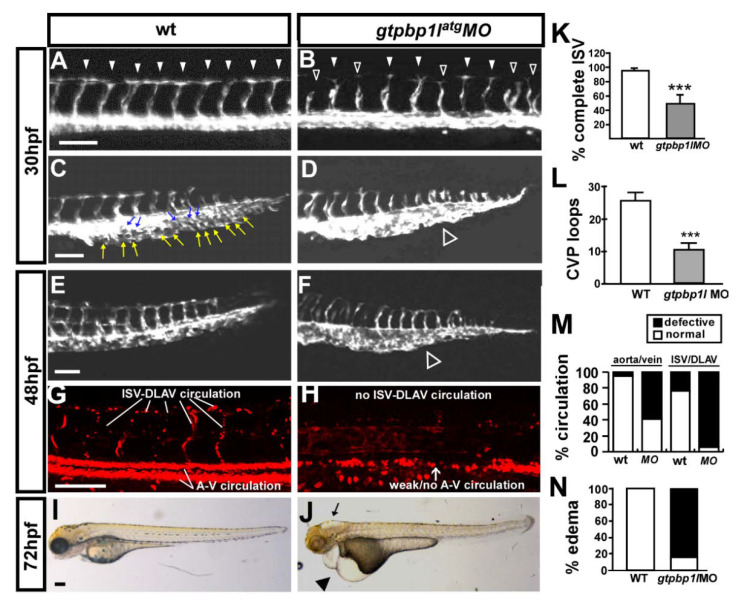
Knockdown of *gtpbp1l* causes vascular defects in zebrafish development. (**A**–**D**) Images of control and *gtpbp1l*^atg^ morpholino (3.4 ng)-injected *Tg (flk:eGFP)* embryos at 30 hpf. In the uninjected control, ISV reached the DLAV at the dorsal aspect of the embryo (arrowheads in (**A**)), and the CVP formed loop structures at the tail ((**C**), white and blue arrows). At the same stage, many ISVs showed slow growth at the mid-somite in *gtpbp1l*^atg^ morphants (hollow arrowheads in (**B**)) and less endothelial cell sprouting and nearly no loop formation at the CVP (white hollow arrowhead in (**D**)). (**E**–**H**) The injection of *gtpbp1l* morpholino into transgenic *Tg* (*fli1a:eGFP^y1^*; *gata1*:*dsRed^sd2^*) embryos showed that knockdown of *gtpbp1l* resulted in circulation defects at 48 hpf. At this stage, there was less CVP loop formation (hollow arrowhead in (**F**)) in MO embryos compared with controls (**E**). (**I**,**J**) At 72 hpf, *gtpbp1l*^atg^ morphants had pericardial edema (arrowhead in (**J**)). (**K**) The percentage of completed ISVs decreased by approximately 40% in *gtpbp1l*^atg^ morphants (*n* = 35 in wt and *n* = 32 in *gtpbp1l*^atg^ MO) at 30 hpf. (**L**) Quantification of CVP formation. (**M**) Quantification data showing that approximately 95% and 60% of *gtpbp1l* morphants at 48 hpf had poor blood flow in ISV–DLAV and in aorta–vein, respectively (*n* = 25 in control and *n* = 23 in *gtpbp1l*^atg^ MO). (**N**) Quantification of the percentage of pericardial edema was 85% in *gtpbp1l*^atg^ morphants (*n* = 26) compared to wt controls (*n* = 30) at 72 hpf. Data are mean ± S.D. *** indicates *p* < 0.0001 by unpaired Student’s *t*-test. Scale bar = 100 µm for (**A**–**H**) and 200 µm for (**I**,**J**).

We took advantage of using transgenic *Tg* (*kdrl*:*eGFP*) zebrafish expressing GFP in endothelial cells to test the potential function of *gtpbp1l* in vascular formation during embryogenesis. We knocked down *gtpbp1l* gene expression by microinjection of 3.4 ng of morpholino (*gtpbp1l^atg^ MO*) to block the ATG translational site. The loss of *gtpbp1l* resulted in two obvious vascular phenotypes, ISV growth defects, and CVP mispatterning (Figure 2A–F,K,L). At 30 hpf, approximately 48% of *gtpbp1l^atg^* morphants had complete ISVs (*n* = 32 embryos) compared to 95% of uninjected control embryos (*n* = 35 embryos). Meanwhile, a secondary phenotype we observed was CVP formation during embryogenesis. At 30 and 48 hpf in the wild-type fish, endothelial cells in the CVP region showed angiogenic sprouting, migration, fusion, and formed honeycomb-like structures or loops (Figure 2C–F). There was less or no honeycomb structure in the CVP in injected embryos compared to the uninjected controls at 30 hpf (Figure 2C,D). At 48 hpf, the swallowed plexus with less or no loops at CVP can be observed and quantified in *gtpbp1l* morphants (*n* = 33) (Figure 2E,F).

Consistent with the vasculature defects in *gtpbp1l* morphants, we found that a loss of *gtpbp1l* resulted in circulation defects and pericardial edema at 48 hpf and 72 hpf. At 48 hpf, we examined the circulation in wild-type and morphants and noted limited blood flow in the trunk of *gtpbp1l 1* morphants. Among *gtpbp1l* morphants, ~40% of *Tg* (*fli1a:eGFP^y1^*; *gata1*:*dsRed^sd2^*) embryos exhibited circulation in the axial vessels, and only ~10% exhibited ISV circulation (Figure 2G,H, *n* = 25 in control and *n* = 23 in *gtpbp1l* ^ATG^ MO) (Figure 2M). At 72 hpf, we found that a loss of *gtpbp1l* caused increased pericardial edema in ~90% of embryos (Figure 2I–N, *n* = 30 in control and *n* = 26 in *gtpbp1l^atg^* MO) (Figure 2N). Edema and circulation defects are common side effects of blood vessel impairment. These data correlated with the vasculature defects in the *gtpbp1l* knockdown embryos.

### 3.3. Specificity of gtpbp1l Knockdown by Morpholino Injection

In Figure 2, we found that knockdown of *gtpbp1l* by inhibiting the translation ATG start site (*gtpbp1l^atg^* MO) impairs vascular development. To demonstrate the phenotypic specificity of *gtpbp1l* morpholino knockdown, we designed a second morpholino (*gtpbp1l^e4i4^* MO) that interferes with *gtpbp1l* pre-RNA splicing at the exon 4-intron 4 boundary and examined the effects in vasculature. Our data showed that the phenotype of vascular defects in *gtpbp1l^e4i4^*MO (Figure S2) fish wasa very similar to those in the *gtpbp1l^atg^* morphants. We observed vascular defects in CVP formation and ISV growth, with a 34% decrease at 30 hpf (Figure S2A–F,I,J). A vascular defect in CVP loops formation was also observed at 48 hpf (Figure S2E,F). We also found edema in *gtpbp1l^e4i4^* MO at 72 hpf (Figure S2G,H,K). We further examined the efficiency of *gtpbp1l^e4i4^* morpholino knockdown by RT–PCR analysis. As the data showed, the injection of 8 ng of *gtpbp1l^e4i4^* morpholino decreased the level and mis-spliced fragments of *gtpbp1l* (Figure S2L,M), indicating the *gtpbp1l^e4i4^* morpholino inhibition of the *gtpbp1l* gene is efficient. These results also confirmed that the vascular defects caused by *gtpbp1l* knockdown are specific.

**Figure 3:**
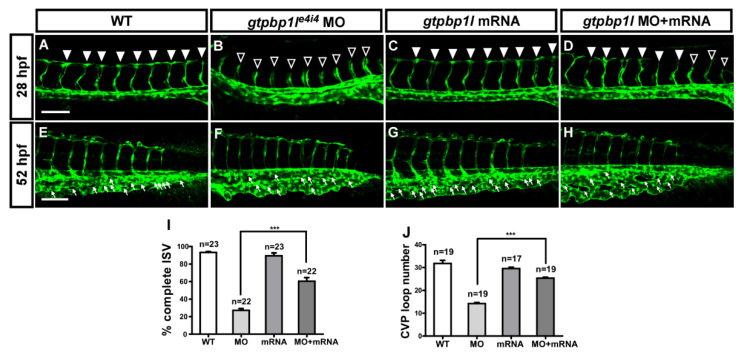
Overexpression of *gtpbp1l* rescues the loss of *gtpbp1l.* (**A**) In wt control embryos, ISV grows toward the dorsal and formed DLAV at approximately 28–30 hpf (*arrowheads*). However, ISVs showed slowed or stalled growth at mid-somite in *gtpbp1l^e4i4^* MO ((**B**), *hollow arrowheads*). (**C**) Overexpression of *gtpbp1l* by low-dosage *gtpbp1l* mRNA injection (0.012 ng) showed no defects in vasculature; however, overexpressed *gtpbp1l* can rescue the growth defect of ISV ((**D**), *solid arrowheads*). (**E**–**H**) At 52 hpf, fewer endothelial cells sprouted, and loop formation occurred at the CVP in *gtpbp1l* MO (*white arrows* in (**F**)) compared to the wt control (**E**). Injection of *gtpbp1l* mRNA induced no obvious defect in CVP (**G**) but rescued the defect of CVP loops (**H**). (**I**) Quantification of the percentage of embryos with completed ISV at 28 hpf shows an increase of ~32% in rescued embryos compared to *gtpbp1l* morphants. The percentages of embryos with completed ISV were ~93 ± 7 (*n* = 23), 28 ± 8 (*n* = 22), 90 ± 12 (*n* = 23), and 60 ± 16 (*n* = 22) in the control, *gtpbp1l* MO knockdown, *gtpbp1l* mRNA overexpression, and rescued embryos, respectively. (**J**) Quantification data of CVP formation were ~27 ± 3 (*n* = 19), 12 ± 3 (*n* = 19), 26 ± 4 (*n* = 17), and 17 ± 3 (*n* = 19) in the control, *gtpbp1l* MO knockdown, *gtpbp1l* mRNA overexpression, and rescued embryos, respectively. *** indicates *p* < 0.0001 by unpaired Student’s *t*-test. Data are mean ± S.D. Scale bar = 100 μm for (**A**–**H**).

We further performed rescue experiments by overexpressing *gtpbp1l* in wild-type and *gtpbp1l^e4i4^* morphant embryos to examine the specificity that vascular defects caused by morpholino knockdown. The overexpression of *gtpbp1l* in wild-type embryos had no obvious effect on vascular ISV and CVP development compared to the wt control (Figure 3A,E,C,G). However, we found that the overexpression of *gtpbp1l* mRNA in *gtpbp1l* MO can restore ISV growth to form DLAV by 36% compared to the knockdown of *gtpbp1l* (*gtpbp1l* MO) at 28 hpf (Figure 3D,B,I). In addition, at 52 hpf, overexpression of *gtpbp1l* mRNA rescued the CVP defect in an additional 47% of embryos compared to *gtpbp1l* MO as the baseline (Figure 3E–H,J). Our data also suggested that general development in other tissues and organs was unaffected since the knockdown of *gtpbp1l* at 24–25 hpf did not alter the expression of *cmlc2* (heart marker), *hbee1* (blood marker), *myoD* (somite marker), *shh* (floor plate marker), or *sox3* (neural marker) (Figure S3A–J). Together, these data suggest that the loss of *gtpbp1l* causes vascular defects that are gene-knockdown-specific and phenotype-specific.

### 3.4. Loss of gtpbp1l Impairs the Growth of ISV Cells

**Figure 4:**
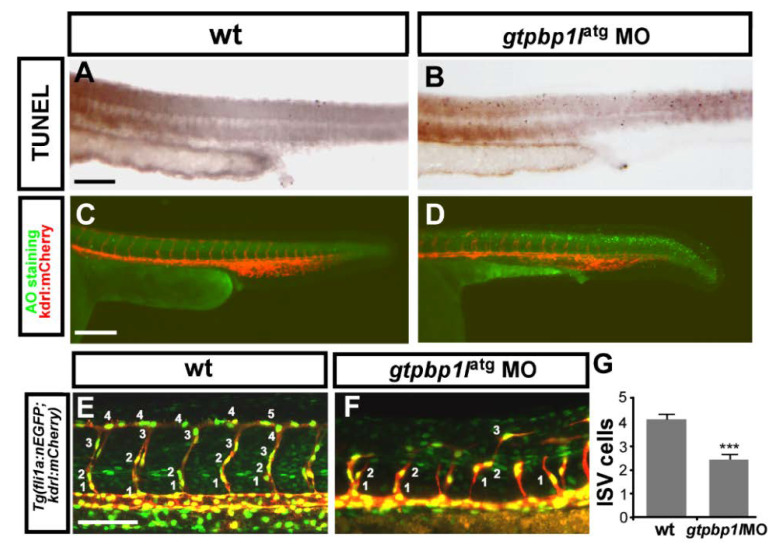
*gtpbp1l* is required for the proliferation and migration of ISV cells. (**A**–**D**) The TUNEL assay and Acridine orange (AO) staining were used to examine apoptotic cells in wild-type (wt) control and *gtpbp1l* knockdown morphants. (**A**,**B**) The number of apoptotic cells (black dots) likely increased in the epidermis of the dorsal region instead of vessel areas in *gtpbp1l* MO compared to controls at 30 hpf. (**C**,**D**) AO staining in *Tg (kdrl:mCherry)^ci5^* fish showed that more apoptotic cells (green dots) were present in the dorsal region of the embryos, but not in vasculature (red fluorescence) after *gtpbp1l* knockdown. (**E**,**F**) At 30 hpf, using transgenic fish *Tg(kdrl:mCherry^ci5^; fli1a:negfp ^y7^)*, the number of ISV endothelial cells can be counted in wt control and *gtpbp1l* morphants. (**G**) Quantification of the average numbers of ISV endothelial cells per ISV in wt control (4.1 ± 0.8, *n* = 30 ISVs from 6 embryos) and *gtpbp1l* morphants (2.5 ± 1.3, *n* = 30 ISVs from 6 embryos). Scale bars are 200 μm for (**A**–**D**) and 100 μm for (**E**,**F**). Data are represented as means ± S.D. *** refers to *p* < 0.0001 by an unpaired Student’s *t*-test.

Loss of *gtpbp1l* results in vascular growth defects, suggesting the growth or survival interruption of endothelial cells. To evaluate whether endothelial cells suffer the problems of proliferation, migration, or cell death, we first examined apoptotic cells by using a TUNEL assay and Acridine orange staining. We showed that *gtpbp1l* morphants have slightly increased apoptosis (apoptotic spots) in the dorsal and epidermal region compared to uninjected wt control embryos at 30hpf. However, morpholino-induced cell death is not present in the vessel region of the trunk by checking vessel *mCherry* signals (Figure 4A–D), indicating that the vascular defect of *gtpbp1l* morphants is not due to the death of endothelial cells. To examine whether the loss of *gtpbp1l* affects cell proliferation, we counted the number of endothelial cells per ISV in the *Tg(kdrl:mCherry;fli1a:negfp ^y7^)* embryos. *gtpbp1l* morphants showed significantly decreased ISV cells compared to the wild-type control (Figure 4E–G, *n* = 30 in *gtpbp1l* morphants and *n* = 30 in wt control). Moreover, fewer ISV cells can migrate to the top of the embryo to form DLAVs, suggesting the interruption of ISV endothelial cell migration in *gtpbp1l* Mos. These data suggested that *gtpbp1l* is critical for endothelial cell growth to form ISVs and CVP, likely via regulating cell proliferation, migration, or both.

### 3.5. Knockdown of gtpbp1l Alters the Expression of Vessel Genes

**Figure 5:**
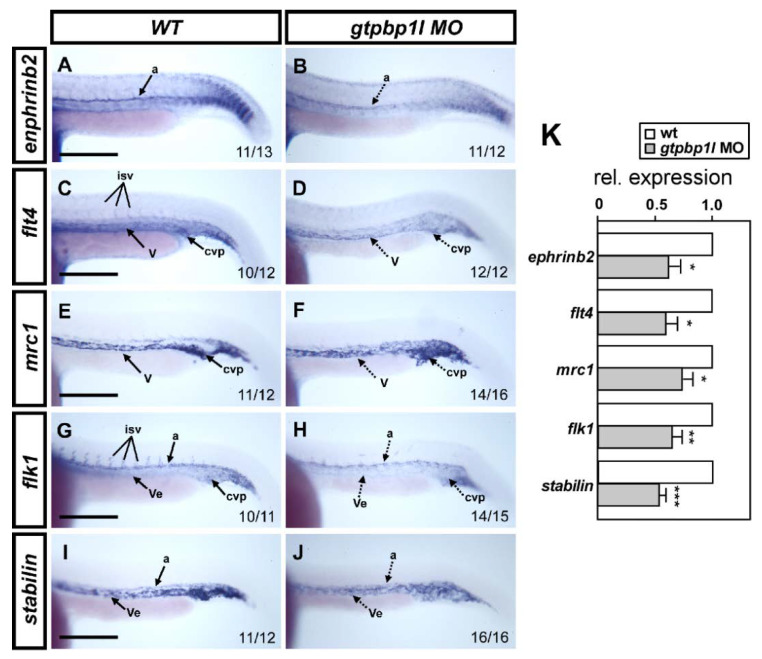
Loss of *gtpbp1l* reduced the expression of vessel-related markers. (**A**,**C**,**E**,**G**,**I**) Whole-mount in situ hybridization in wild-type (wt) controls and in *gtpbp1l*^atg^ morphants (**B**,**D**,**F**,**H**,**J**). At 24 hpf, *gtpbp1l*^atg^ morphants showed decreased expression (dash lines) of the arterial marker *ephrinb2* (**B**), venous markers *flt4* (**D**) and *mrc1* (**F**), and pan-vascular markers *flk* (**H**) and *stabilin* (**J**) compared to controls. Dorsal aorta (a); vein (v); vessel (Ve); intersegmental vessels (isv), and caudal vein plexus (CVP). (**K**) Quantitative qPCR analysis revealed the relative expression levels of *ephrinb2* (0.63 ± 0.22), *flt4* (0.60 ± 0.22), *mrc1*(0.74 ± 0.25), *flk1*(0.63 ± 0.20), and *stabilin* (0.54 ± 0.12) in *gtpbp1l*^atg^ morphants, which is normalized to wt controls. Values on the bottom right indicate the number of embryos exhibiting the staining pattern per total number. Data are mean ± S.D. *** *p* < 0.0001, ** *p* < 0.001, and * *p* < 0.05 according to unpaired Student’s *t*-test. Scale bars are 200 µm.

The observed vascular defects in ISV and CVP suggest *gtpbp1l’s* role in the vasculature to modulate vascular identity. To determine how it is affecting vascular markers, we checked their expression via in situ hybridization and qPCR. We examined typical vascular markers including *flk, flt4, mrc1, stabilin,* and *ephrinb2* at 24 hpf. We noted the expression of the *flk* and *stabilin* (pan-vascular markers), *flt4* (venous/ISV marker), and *mrc1* (venous marker) was reduced significantly in the *gtpbp1l* knockdown group compared to wild-type controls (Figure 5). The relative expression of these markers shows a reduction of approximately 30–50% in their expression in the knockdown group. These results indicate *gtpbp1l’s* role in the regulation of vascular genes to control the development of healthy vessels.

### 3.6. Interaction between gtpbp1l and Multiple Signals

**Figure 6:**
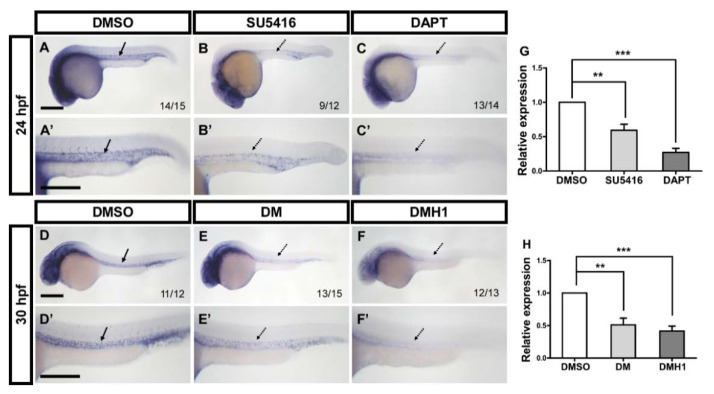
Interactions among *gtpbp1l*, VEGF/Notch, and BMP signals. (**A**–**C**) At 24 hpf, the expression level of *gtpbp1l* was decreased in SU5416-treated (**B**) and DAPT-treated embryos (**C**) compared to DMSO-treated control embryos. (**D**–**F**) At 30 hpf, inhibition of BMP signals by DM or DMH1 treatment reduced the expression of *gtpbp1l* in embryos compared with that in DMSO-treated control embryos. (**A’**–**F’**) Enlargements of (**A**–**F**). (**G**) The relative expression level was quantified by the qPCR assay and showed a significantly decreased expression of *gtpbp1l* in SU5416-treated (0.59 ± 0.23) and DAPT-treated (0.27 ± 0.12) embryos compared to controls. (**H**) Quantification of the relative expression level by qPCR analysis in DM-treated (0.51 ± 0.23) and DMH1-treated (0.42 ± 0.19) embryos had the reduced expression of *gtpbp1l*, which is normalized to DMSO-treated controls. Values on the bottom right indicate the number of embryos exhibiting the phenotype per total number of embryos analyzed. Data are represented as means ± S.D. *** refers to *p* < 0.0001 and ** *p* < 0.001 by unpaired Student’s *t*-test. Scale bars are 200 µm.

Our data showed that the loss of *gtpbp1l* interrupted the growth and patterning of ISVs and CVP. Notch signaling is crucial for the specification of venous cells and ISV tip cells and important for the migration and proliferation of endothelial progenitor cells via the interaction with VEGFR2 signaling during angiogenesis in the zebrafish trunk. Therefore, we tested the regulatory relationships between *gtpbp1l* and *Notch* or *VEGFR2*. We inhibited Notch and VEGFR2 signaling through exogenous DAPT and SU5416 treatment, respectively (Figure 6A–D). We found that the expression of *gtpbp1l* was downregulated when Notch or VEGFR2 signals were inactivated. These data suggest that *gtpbp1l* controls vascular patterning and is likely regulated by the Notch and VEGF pathways.

In addition, BMP signaling was reported to regulate angiogenic sprouting from the axial vein to pattern the CVP, which provides a different regulating mechanism of angiogenesis from ISV growth [12,13]. We therefore tested whether *gtpbp1l* interacts with BMP signaling to function in CVP formation by using BMP signaling inhibitors DMH1 and DM. Compared with DMSO treatment as a control, DM or DMH1 treatment reduced the expression level of *gtpbp1l* in the embryos (Figure 6E–H). These data suggest that *gtpbp1l* is downregulated by BMP signaling. Collectively, these results suggest that *gtpbp1l* controls ISV and CVP through VEGF/Notch and BMP signaling.

## 4. Discussion

GTPase proteins have been shown to be involved in many important biological processes. They encompass a large group of enzymes that bind GTP and undergo a conformational change as GTP is hydrolyzed to GDP. The main groups of GTPases are heterotrimeric G-proteins (G-coupled receptors, GPCRs) and small GTPases (including several superfamilies, e.g., Ras, Rho/Rac/Cdc42, Rap, Ran, and Arf) [25,26,27]. The coordinated activation of Rho GTPases by signals is required for angiogenic processes, including cell guidance, migration, proliferation, and morphogenesis [28]. The regulation of Rho GTPase activation is mediated by interactions with guanine nucleotide exchange factors (GEFs) or GTPase-activating proteins (GAPs). Many GEFs have been shown to regulate angiogenesis and vascular function by fine-tuning the actions of Rho GTPase [26]. Vascular malformations are linked to mutations in RAS p21 protein activator 1 (Rasa1 or p120RasGAP). Kawassaki et al. showed that Rasa1/Ras interacts with the Ephb4 signaling pathway to suppress endothelial mTORC1 activity and Rasa1 mutation leads to capillary malformation arteriovenous malformations (CM-AVMs) [29]. In addition, recent transcriptome analysis reveals molecular signatures in cerebral cavernous malformation (CCM) endothelial cells, which provides the fundamental role of growth factors and RAS in the field of pathologies related to angiogenesis [30].

Many translational GTPases (trGTPases) are essential for life due to their pivotal roles in the translation cycle on the ribosome. The classical trGTPases (IF2, EF-Tu, EF-G) are highly conserved and well-studied in the function of the translation process. GTP-binding proteins (GTPBPs) comprise a divergent group of translational GTPases with uncertain functions but are closely related to EF1A and RF3 [31]. Although GTPBPs have widespread expression, physiological functions remain limited. Initial studies focused on their tissue specificity, suggesting the role of GTPBP1 and GTPBP2 in innate immunity [32,33]. Recent studies revealed the different biochemical roles of GTPBP1 and GTPBP2 [17,18]. GTPBP1 likely directs the exosome to mRNA targets. GTPBP2 can interact with aa-tRNA but lacks elongation activity and does not promote exosome degradation, suggesting that GTPBP1 and GTPBP2 have different roles. Terrey et al. found that GTPBP1 resolves paused ribosomes to maintain neuronal homeostasis linked to mTOR signals [19]. A study on GTPBP2 showed that ribosome stalling induced by the mutation of a CNS-specific tRNA causes neurological defects [34]. The essential role of *Gtpbp2* in neural homeostasis was further revealed by the fact that mutations in *Gtpbp2* in humans cause neurodegeneration and intellectual disabilities [34,35]. In addition, Gtpbp2 has been shown to interact with Smad1/BMP signaling and is essential for mesodermal development in *Xenopus* embryos [36]. Knockout of Gtpbp3 in zebrafish caused hypertrophic cardiomyopathy and showed abnormal mitochondrial tRNA metabolism [37]. In this study, we reported a novel function of the GTPBP superfamily in vascular development, identified in this first investigation of the role of GTPBP in vasculature. We also revealed that *gtpbp1l* regulates vascular development mediated by VEGF/Notch and BMP signals.

Based on the known biochemical function of GTPBPs, we propose two potential mechanisms by which gtpbp1l regulates vascular development. First, *Gtpbp1l* is important for vessel growth mediated by proangiogenic protein synthesis, i.e., Gtpbp1l possesses eEF1A-like elongation activity, delivering cognate aminoacyl-transfer RNA (aa-tRNA) to the ribosomal A site in a GTP-dependent manner, supporting the protein synthesis of angiogenesis or angiogenic signals. Second, Gtpbp1l regulates vessel growth mediated by exosome-dependent mRNA degradation of antiangiogenic factors. Similar to AANAT (aralkylamine N-acetyltransferase), mRNA turnover is controlled by the interaction between GTPBP1 and exosomes [18]. Thus, GTPBP1l may act as a regulator and adaptor of the exosome-mediated mRNA turnover pathway. In addition, exosomes (extracellular vesicles) have been shown to carry microRNAs or ligands (VEGF, PDGF, etc.) that are important for angiogenesis; thus, the disruption of exosome trafficking, docking, or release might impair vascular growth [38]. Xu et al. showed that neurons secrete miR-132-containing exosomes to regulate brain vascular integrity and miR132 knockdown impairs vascular integrity in zebrafish [39].

It has been reported that Gtpbp2 interacts directly with Smad1 to regulate BMP signaling and is essential for mesoderm development in a frog model. How *gtpbp1l* interacts with VEGF or BMP signals will be interesting to address. The translation is highly regulated by two signal pathways: The mTOR signaling and the integrated stress response (ISR) [40]. Whether gtpbp1l regulates vessel growth mediated by mTOR signals is also intriguing. Finally, a mammalian study suggested functional redundancy between *Gtpbp1* and *Gtpbp2* [33]. Zebrafish *Gtpbp1* is expressed in the blood during embryogenesis, suggesting its function in blood development. Whether GTPBP1 has vascular function is not known and will be interesting to address.

## 5. Conclusions

In this study, we report a novel gene, *gtpbp1l (GTP-binding protein 1-like),* that controls vascular development in zebrafish. We showed that *gtpbp1l* mRNA is expressed in the vasculature, and the knockdown of *gtpbp1l* impairs the growth of ISVs and the CVP. We further showed vascular defects are likely caused by the impairment of migration and proliferation. We also revealed that *gtpbp1l* is regulated by VEGF and BMP signals. These findings showed that *gtpbp1l* plays a critical role in vascular development. To our knowledge, the novel function of *gtpbp1l* in the genetic network during vascular development has never been explored.

## Data Availability

Not applicable.

## Supplementary Materials

The following supporting information can be downloaded at: https://www.mdpi.com/article/10.3390/biomedicines10123208/s1, Table S1: qPCR primer sequences used in this study; Figure S1: Sequence comparison of GTPBP1l among vertebrates; Figure S2: The knockdown of *gtpbp1l* by splicing MO caused vascular defects; Figure S3: Gross developmental process is unaffected in *gtpbp1l^atg^* morphants.
