# Supplementary material for: GTP-Binding Protein 1-Like (GTPBP1l) Regulates Vascular Patterning during Zebrafish Development
Source: Biomedicines. 2022 Dec 10;10(12):3208. doi: 10.3390/biomedicines10123208 (PMC9775176; doi:10.3390/biomedicines10123208)
Supplement: Supplementary file 1 [file biomedicines-10-03208-s001.zip › biomedicines-2050641-supplementary.pdf]

biomedicines

Supplementary Materials

GTP-binding protein 1-like (GTPBP1l) regulates vascular patterning during zebrafish development

Yi-Hao Lo [1,2,3,†], Yi-Shan Huang [4,5,†], Yu-Chiuan Chang [6,†], Pei-Yu Hung [4], Wen-Der Wang [7], Wangta Liu [8], Ritesh Urade [4], Zhi-Hong Wen [2,3,5] and Chang-Yi Wu [3,4,5,8,*]

Supplementary Methods:

Morpholino efficiency

The efficiency of morpholinos that cause mis-splicing or reduced band signals was determined using polymerase chain reaction (PCR) with primers flanking on exon3 and exon5 (sFig. 2L). RNAs extracted from splicing morpholinos injected and uninjected embryos and reverse transcript to cDNA. Primers *gtpbp1l* MO_f :5′-TCCCTGTGACTTCTGCACTG-3′ and *gtpbp1l* MO_r: 5′-CCTCTGACCTCATCCTCTCG-3′ are used to examine *gtpbp1l* knockdown efficiency, while primers GAPDH_f :5′-TGCTGTAACCGAACTCATTGTC-3′ and GAPDH_r :5′- CAAGCTTACTGGTATGGCCTTC-3′ used as a loading control.

Table S1: qPCR primer sequences used in this study

qPCR primers	Sequence
β-actin_qf	5′-CTCTTCCAGCCTTCCTTCCT-3′
β-actin_qr	5′-CTTCTGCATACGGTCAGCAA-3′
flt4_f1	5′-ACTCGGGTTATTACCGCTGCTTCT-3′
flt4_r1	5′-TGGATGCTCTGGGTCTCGAACAAA-3′
flk1_qf	5′-ACTTTGAGTGGGAGTTTCATAAGGA-3′
flk1_qr	5′-TTGGACCGGTGTGGTGCTA-3′
mrc1_qf	5′-CTAGCAAGCCTGAAGGTGCC-3′
mrc1_qr	5′-TGAGAGGCTGGGTAGTTGGG-3′
ephrinb2_qf	5′-CTGGAACACCACGAACACC-3′
ephrinb2_qr	5′-CACACGTGGGCAAACTATGT-3′
stabilin_qf	5′-GGGCTTCCAATACCAACTGG-3′
stabilin-qf	5′-CCTGGTTGCACAGACAGACC-3′
gtpbp1l_qf	5′-CATCCGACGACCATTTCACC-3′
gtpbp1l_qr	5′-GGCCTGAGGTTTGCTTTGAA-3′

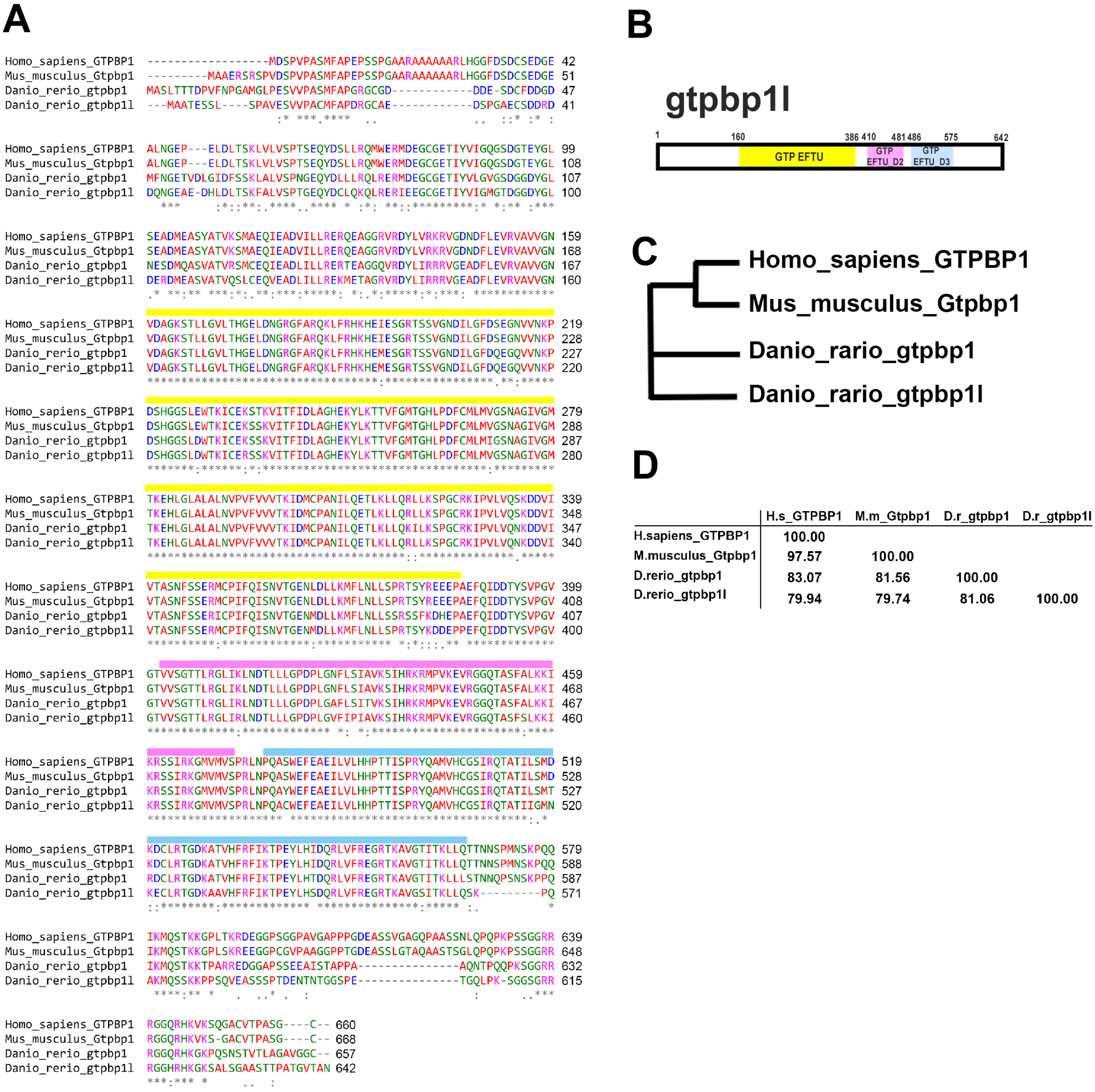

	H.s_GTPBP1	M.m_Gtpbp1	D.r_gtpbp1	D.r_gtpbp1l
H.sapiens_GTPBP1	100.00			
M.musculus_Gtpbp1	97.57	100.00		
D.rerio_gtpbp1	83.07	81.56	100.00	
D.rerio_gtpbp1l	79.94	79.74	81.06	100.00

Figure S1. Sequence comparison of GTPBP1l among vertebrates.
(A) Comparing the amino acid sequences of zebrafish *(D. rerio)* gtpbp1l (NP_001019982.1) to gtpbp1 of zebrafish (NP_998640.2), human (*H. sapiens*, GenBank accession number NP_004277.2) and mouse (*M.musculus,* GenBank accession number NP_038846.2) by using the Clustal Omega software. Identical amino acids are marked (*) below the sequence. The yellow bar, red bar, and blue bar are the conserved functional regions on gtpbp1l, representing a different binding site of GTP prokaryotic elongation factor. **(B)** Schematic diagram of gtpbp1l protein contains the 642 amino acid and estimate 71 kDalton, with three putative GTPase domains related to elongation factor (EF-Tu). **(C)** Amino acid sequence comparison of *gtpbp1-like* and *gtpbp1* among vertebrates, similarity is over 79%. **(D)** The percentage of amino acid sequence alignment of gtpbp1l homologous genes among different species.

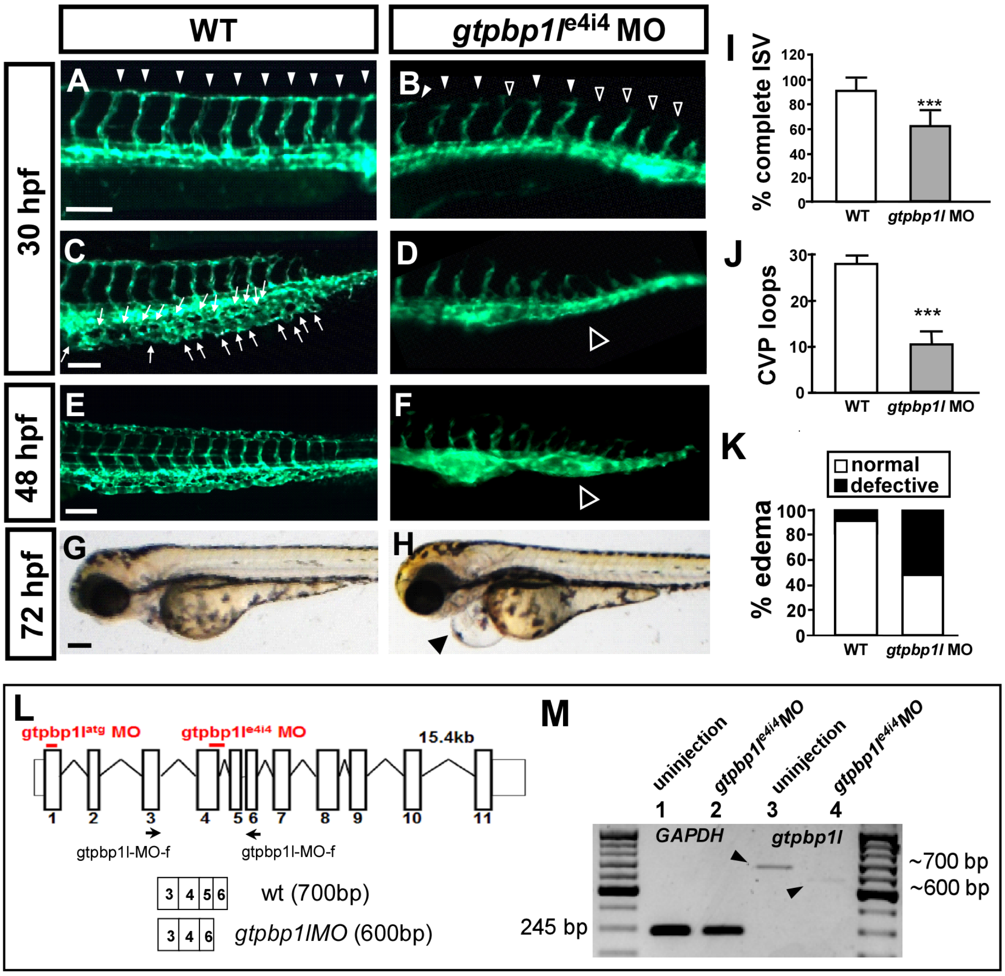

Figure S2. The knockdown of *gtpbp1l* by splicing MO caused vascular defects.
(A–H) The knockdown of *gtpbp1l* by splicing MO caused similar defects in the vasculature, including defects in ISV growth *(hollow arrowheads* in **B***)* and less CVP sprouting and loop formation **(D, F)** *(arrowhead)* compared to wild-type control. **(G, H)** At 72 hpf, *gtpbp1l* ^{e4i4} morphants had pericardial edema (arrowhead in H) and **(K)** quantification data showed 53% edema fish. **(I)** The percentage of completed ISVs decreased about 33% in *gtpbp1l* ^{e4i4} morphants (n = 23 in wt and n = 25 in *gtpbp1l* ^{e4i4} MO) at 30 hpf. **(J)** The loop formation in CVP exhibited a decreased number (10.1 ± 3.8**)** in *gtpbp1l* ^{e4i4} morphants compared to wt control (27.2 ± 2.8) (n = 20 in wt and *gtpbp1l* ^{e4i4} MO) at 48 hpf. Data are represented as means ± S.D. *** refers to p < 0.0001 according to the unpaired Student's *t*-test. Scale bars are 100 μm for **A-F** and 200 μm for **G, H**. **(L)** The scheme presents *gtpbp1l^{e4i4}* morpholino targeting the pre-mRNA structure of *gtpbp1l*, suggesting the missplicing of a fragment, which can be detected using a *gtpbp1l* mo_f and *gtpbp1l gtpbp1l*mo_r primer set. **(M)** Total RNA from controls or *gtpbp1l* morphants (injected with 8.0 ng *gtpbp1l^{e4i4}* morpholino) were used in RT-PCR with primers for the *gtpbp1l* gene. In morphants injected with *gtpbp1l^{e4i4}* morpholino, GAPDH levels remain unchanged (245bp, lane 1-2), whereas the amount of *gtpbp1l* product (~600bp) mis-spliced and decreased compared to normal splicing product (~700bp).

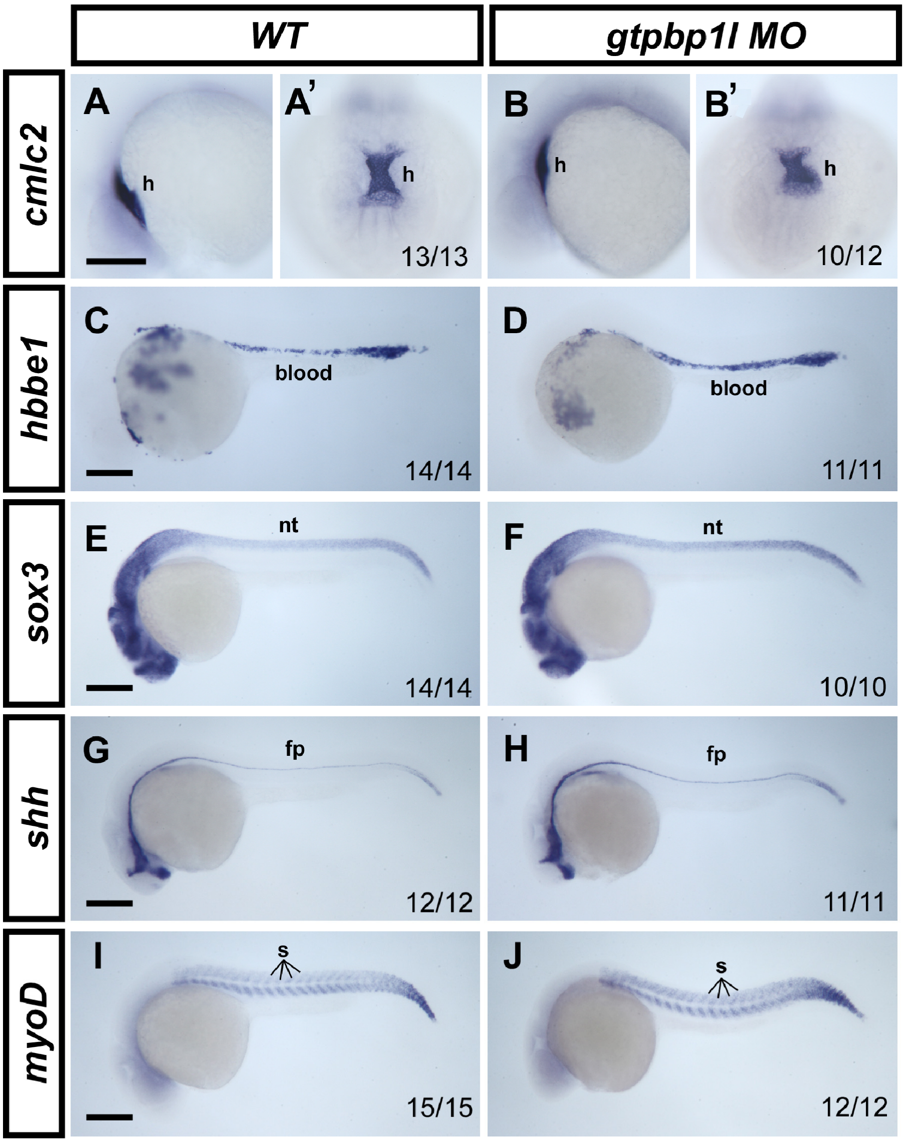

Figure S3. Gross developmental process is unaffected in *gtpbp1l*^{atg} morphants.
Knockdown of *gtpbp1l* did not alter the expression pattern of heart, somite and neural systems by examining the expression of *cmlc2* (heart marker, h; **A-B** are lateral view and **A'-B'** are ventral view), *hbbe1* (blood marker, **C-D**), *sox3* (neural tube marker, nt, **E, F**), *shh* (floor plate, fp, **G, H**) and *myoD* (somite marker, s, **I, J**). Those probes have been described and documented in ZFIN. Values on the bottom indicate the number of embryos exhibiting phenotype per total number of embryos analyzed. Scale bars in all figures are 200 μm.
